# Probiotic Yogurt Fortified with Chickpea Flour: Physico-Chemical Properties and Probiotic Survival during Storage and Simulated Gastrointestinal Transit

**DOI:** 10.3390/foods9091144

**Published:** 2020-08-19

**Authors:** Manwinder Kaur Sidhu, Fengzhi Lyu, Thomas Patrick Sharkie, Said Ajlouni, Chaminda Senaka Ranadheera

**Affiliations:** School of Agriculture & Food, Faculty of Veterinary and Agricultural Sciences, The University of Melbourne, Parkville, VIC 3010, Australia; manwinderkaur91@yahoo.com (M.K.S.); fengzhil@student.unimelb.edu.au (F.L.); tsharkie@student.unimelb.edu.au (T.P.S.); said@unimelb.edu.au (S.A.)

**Keywords:** dairy, yogurt, chickpea flour, probiotics, lactic acid bacteria

## Abstract

In the present study, probiotic yogurt with *Lactobacillus acidophilus* LA5 and *Bifidobacterium* BB12 was produced via fortification with chickpea flour (0, 1, 2.5, 5% *w/v*). During refrigerated storage for five weeks, probiotics maintained a viable count above the minimum therapeutic level (10^6^ CFU/g) in all yogurt types. Although there was no significant (*p* > 0.05) positive effect of chickpea flour on probiotic viability during storage, the addition of chickpea flour has beneficial effects on the viability of both probiotic species in the presence of gastric and intestinal juices, with 0.3% bile. This study also evaluated the physio-chemical properties of probiotic yogurt during storage. Some physicochemical properties of yogurt, such as water holding capacity and susceptibility to syneresis, were enhanced by the addition of chickpea flour. Hence, chickpea flour could be an attractive pulse ingredient in the production of probiotic yogurts for health-conscious consumers.

## 1. Introduction

Probiotics are live microorganisms, which confer health benefits on the host, when administered in adequate amounts [1], such as maintaining a healthy gut microbiome and competing with pathogenic microorganisms [2]. Various species of bacteria have been used as probiotics. Among those, species of *Lactobacillus* and *Bifidobacterium* have been widely used and are considered safe and effective [3,4]. Fermented dairy products, such as yogurts, are considered to be the main vehicle for delivering beneficial probiotic bacteria to humans [5]. Yogurt is formed through the coagulation of milk protein by lactic acid, which is produced by lactic acid bacteria in the starter culture [6]. Yogurt is not only highly recommended for those suffering from gastro-intestinal disorders and lactose intolerance, but is beneficial to boost the immune system [3,4,7]. Moreover, yogurts are also used as a vehicle for the delivery of several key nutrients such as protein, minerals and various micronutrients [8]. Therefore, these products are becoming increasingly popular among health-conscious consumers. Fruit juices, legumes, cereals and dry fruits are increasingly used to fortify yogurt [8,9,10]. It is difficult to produce yogurt with a good consistency using only probiotic cultures, without starter cultures such as *Lactobacillus delbrueckii* subsp. *bulgaricus* and *Streptococcus thermophilus* [11]. Generally, in yogurt production, fermentation needs to be carried out with these lactic acid producing microorganisms. The slow growth of probiotics may also permit the growth of undesirable organisms [11]. Starter cultures can help to maintain the viability of probiotics by providing favorable metabolic substances for certain probiotics such as *Bifidobacterium*. Therefore, in probiotic yogurt manufacture, both probiotics and starter cultures are used together as adjunct cultures. Starter culture bacteria are also known to provide benefits and enhance consumers’ immune system [12]. However, their gastrointestinal survival is poor and hence use of the term probiotics to describe starter cultures is controversial [13]. To provide health benefits, probiotics should survive both in the food matrix during storage and through gastrointestinal transit after consumption. They must also reach the large intestine in sufficient numbers. Hence, probiotics should maintain a minimum viability level of 10^6^–10^7^ CFU/mL or g of carrier food product, at the time of consumption. This concentration is known as the minimum therapeutic level [4,14]. Effective probiotic microorganisms should tolerate harsh gastric and intestinal conditions including acid, bile and digestive enzymes, as well as have the ability to attach to the gut epithelium to produce desired health benefits [4,15].

The viability of probiotics in yogurt is affected by many factors such as the oxygen content of the product and oxygen permeation through packaging materials [16]. The viability of probiotics in yogurt is also affected by the concentration of lactic acid, hydrogen peroxide and bacteriocins which are mainly produced by the starter cultures [17]. Hence, maintaining the minimum therapeutic level of probiotics in yogurt during storage is a challenge. Consequently, incorporation of prebiotics in such products is one of the major strategies to enhance probiotic viability. Prebiotics are non-digestible food ingredients that promote the growth and activity of beneficial microorganisms [18]. Certain plant-derived foods, including pulses, are abundant in prebiotics [19].

Pulse flour is becoming increasingly popular in extending or substituting animal-derived products, due to recent interest in sustainable food production and the conferred health benefits [20,21]. For example, pea flour is highly digestible and contains a high amino acid content including high levels of lysine and arginine [20]. Due to its high fiber and protein content, pea flour is also considered to be a potential prebiotic for probiotic species of *Lactobacillus* [19]. Chickpea (*Cicer arietinum* L.) is rich in protein, fiber and other prebiotic substances. As a food ingredient, it may enhance the nutritional and functional qualities of food products. Hence, chickpea flour may be a highly attractive substance to incorporate into the formulation of yogurt. Recent studies have demonstrated successful incorporation of chickpea flour into yogurt [22]. Although many of these studies have focused on probiotic viability, physicochemical and sensory properties, studies examining the gastrointestinal survival of probiotic lactobacilli and bifidobacteria in chickpea-fortified yogurt are limited. This study aimed to produce chickpea flour-enriched yogurt containing probiotic *Lactobacillus acidophilus* LA5 and *Bifidobacterium* BB12 and evaluate the potential of the yogurt to confer enhanced in vitro gastrointestinal tolerance of these probiotics. Product quality characteristics, including probiotic viability over five weeks of refrigerated storage, and major physicochemical properties were also evaluated.

## 2. Materials and Methods

### 2.1. Preparation of Yogurt Fortified with Chickpea Flour

Skim milk powder (Woolworths, Australia) was added to cow’s milk (Woolworths, Australia) at a concentration of 1%, before pasteurization at 80–85 °C for 30 min. The pasteurized milk mixture was cooled to 40–42 °C before adding 0.2 g/L of freeze-dried ABY-10 yogurt culture containing both starter cultures (*Streptococcus thermophilus* and *Lactobacillus delbrueckii* subsp. *bulgaricus*). The probiotic *Lactobacillus acidophilus* LA5 and *Bifidobacterium* BB12 were also added according to manufacturer’s recommendations (Christian Hansen, Australia). Plain yogurt with no added chickpea flour was produced by placing the inoculated milk in a 250 mL sterile plastic container and incubating at 42 ± 1 °C until pH 4.4–4.5 was reached. Similarly, another three batches of yogurt, each 250 mL, were produced by incorporating 1, 2.5 and 5% (*w/v*) roasted chickpea flour (McKenzie’s Foods, Melbourne, VIC, Australia). The flour was roasted at 60 °C for 10–15 min. After combining flour with the yogurt, it was incubated under the same conditions as the plain yogurt. All types of yogurt were stored at 4 °C for 5 weeks. Yogurt production was repeated three times. According to the manufacturer, the commercial chickpea flour contained nutrients in the following approximate composition: energy, 1510 kJ; protein, 21.8 g; fat, total 6.0 g; -saturated, less than 1 g; carbohydrate, 45.7 g; -sugars, 2.6 g; dietary fiber, 13.4 g; sodium, less than 5 mg; gluten—nil.

### 2.2. Analysis of Physicochemical Properties

The pH of yogurt samples was measured by using a digital pH meter (HANNA Instruments; Smithfield, RI, USA). The titratable acidity of yogurt samples was measured by titrating 9 g of yogurt sample with 0.1 NaOH solution and using phenolphthalein (Chem-supply, Gillman, SA, Australia) as an indicator [23]. The total solids were determined by drying samples at 105 ± 1 °C overnight to constant weight. The ash content was measured by ignition of solid materials at 550 °C in an electric muffle furnace (Tetlow Furnaces, VIC, Australia). Susceptibility to syneresis was measured by placing 50 mL of each yogurt sample in a funnel lined with Whatman filter paper number 1 (Whatman International Ltd., Maidstone, England). After 6 h of drainage, the volume of whey collected in the beaker was measured and the susceptibility to syneresis was calculated as per Ranadheera et al. [23]. Water holding capacity of the yogurt samples was measured through centrifugation [23]. Viscosity of the sample was measured in centipoises with the use of a digital viscometer, model DV-2 + Pro (Brookfield Engineering Laboratories, Middleboro, MA, USA) and spindle number LV 7 rotated at 10 rpm. The color of each yogurt sample was measured by using a colorimeter (CR-410 Chroma Meter; Konica Minolta, Ramsey, NJ, USA). All properties were measured in triplicate.

### 2.3. Microbiological Analysis: Viability of Probiotics and Starter Cultures in Yogurt during Storage

Microbial viability in yogurt was determined using serial dilution and spread plating techniques and expressed as CFU/g [23]. Media were purchased from Edwards (Narellan, NSW, Australia) and Thermo Fisher Scientific Australia Pty. Ltd. (Scoresby, VIC, Australia). De Man–Rogosa–Sharpe (MRS) bile agar (0.2 g of bovine bile in 1 L of MRS agar) was used for the selective enumeration/counting of *L. acidophilus* LA5 in yogurt samples. pH-modified (4.58) MRS agar was used for enumeration/counting of *L. delbrueckii* supsp. *bulgaricus*. M17 agar was used for enumeration/counting of *S. thermophilus* [23]. Reinforced clostridia agar (RCA) with aniline blue and dicloxacillin was used for selective enumeration/counting of *Bifidobacterium* BB12. All plates were incubated for 48–72 h under anaerobic conditions at 37 °C except *S. thermophilus,* which was incubated under aerobic conditions. In addition to the use of the above selective media, macroscopic observations and Gram-staining have been performed to identify and confirm each microbial species grown on each selective medium in this study.

### 2.4. Preparation of Simulated Gastric and Intestinal Juices

Simulated gastric juices were prepared by suspending pepsin (Thermo Fisher Scientific, Scoresby, VIC, Australia) in 0.5% (*w/v*) saline solution to reach a final concentration of 3 g/L. Then, the pH of the solution was adjusted to 2.0 by adding concentrated HCl. Simulated small intestinal juices were prepared by suspending pancreatin (Thermo Fisher Scientific, Scoresby, VIC, Australia) in sterile 0.5% (*w/v*) saline solution to a final concentration 1 g/L, with or without 0.3% bile salts (Sigma-Aldrich, Castle Hill, NSW, Australia). NaOH (0.1 M) was used to adjust the pH to 8.0.

### 2.5. In Vitro Gastro-Intestinal Tolerance and Probiotic Survival

In 15 mL polypropylene tubes (Falcon, Mexico), 9 mL of gastric juices or intestinal juices and 1 g of yogurt (0, 1, 2.5, 5% chickpea flour) were mixed for 10 s using a vortex mixer at maximum setting (VM1, Ratek Instruments Pty. Ltd, Boronia, VIC, Australia). The samples were incubated at 37 °C. Aliquots of 1 mL were removed to assess resistance to gastric juices (after 1-, 60- and 180-min exposure) and to intestinal juices with or without 0.3% bile (after 1-, 60- and 240-min exposure) [10]. Serial dilutions and spread plating were then performed to determine total viable counts at each time points as described in Section 2.3. All assays were conducted in duplicate.

### 2.6. Statistical Analysis

Repeated measures analysis of variance was used for analysis of microbial viability and gastrointestinal survival. Physiochemical properties were analyzed using one-way analysis of variance. The means were separated using the least significant difference at a 95% confidence level.

## 3. Results and Discussion

### 3.1. Physicochemical Properties of Yogurt

Yogurt pH and acidity are important parameters that determine product quality. Yogurt pH values in all samples in the present study were generally within the desirable range (~4.0–4.5) as suggested by previous studies [24]. As predicted, titratable acidity (TA) was negatively correlated with pH value (Table 1). In general, the pH of chickpea flour is around 6.4 [25]. The main driver of acidity development in yogurt is the fermentation of milk by lactic acid bacteria [26], such as *L. delbrueckii* subsp. *bulgaricus* [24], *S. thermophilus*, *L. acidophilus* and *Bifidobacterium* spp. [27]. Lactose in milk is converted into lactic acid by a starter culture and probiotic bacteria during fermentation [28], which leads to the increased acidity of the yogurt. The yogurt products generated in this study employ both a starter culture and probiotic bacteria to generate acceptable TA and pH. Higher acidity development in yogurts with 2.5 and 5% chickpea flour may be attributed to the possible growth promotion prebiotic effect of chickpea flour towards probiotics and starter cultures during yogurt fermentation.

As shown in Table 1, the higher percentage composition of chickpea flour (≥2.5%) significantly increased the water holding capacity (WHC) of yogurt (*p* < 0.05). Several studies have reported that the WHC of yogurt is mainly related to the ability of protein and fat globules to retain water [29]. Chickpea flour has a high content of fat and protein, which was also indicated by higher levels of total solids and ash contents in yogurt samples with higher chickpea flour (Table 1). The composition of chickpea flour, therefore, could have contributed to the enhanced WHC of the yogurt seen in this study. The viscosity of the yogurt was also affected by the addition of chickpea flour. Viscosity levels became significantly higher as more chickpea flour was incorporated into the yogurt formulation (Table 1). Syneresis is regarded as an important indicator of yogurt quality and, in general, higher syneresis indicates inferior product quality. Usually, syneresis is negatively correlated with the WHC of yogurt. In this study, data showed that the susceptibility of yogurt to syneresis decreases as the concentration of chickpea flour increases (Table 1). This could be mainly attributed to the presence of a high concentration of protein, fiber and fat in legumes [30]. Yousseef et al. [20] have reported similar observations around the alleviation of the syneresis in yogurt containing high concentrations of pea flour. A comparison of the L *, a * and b * values for color between the different yogurt formulations during storage is shown in Table 2. There was no significant difference (*p* > 0.05) in L * value across the four formulations. Noh et al. [31] drew a similar conclusion that supplementation of yogurt with extracts of *Corni fructus* did not significantly affect the L * value. However, contrary results have been reported by Costa et al. [32] that this color parameter was dramatically influenced by the addition of probiotics and prebiotic ingredients. The whiteness of milk is due to the presence of fat globules and casein micelles, which are capable of scattering white light in the visible spectrum [33]. Thus, the difference observed in the L * value may be due to variations in the milk composition and prebiotics selected. Moreover, due to the fact that chickpea flour is visually yellow-green in color, all yogurts fortified with chickpea flour were found to be greener (as indicated by negative *a* * values), while a greater yellow coloration as indicated by the b * value only occurred under a high concentration of the flour (≥2.5%).

### 3.2. Viability of Probiotics and Yogurt Starters during Storage

**Figure 1:**
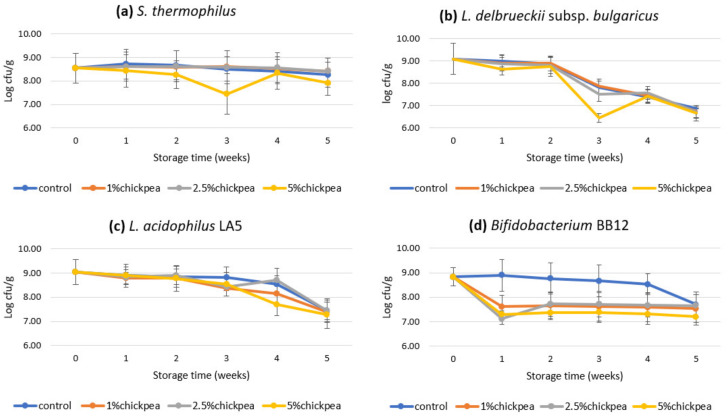
Viability of (**a**) *S. thermophilus*, (**b**) *L. delbrueckii* subsp. *bulgaricus*, (**c**) *L. acidophilus* LA5 and (**d**) *Bifidobacterium* BB12 in fortified yogurt with chickpea flour during 5-week storage at 4 °C (mean ± SD).

As shown in Figure 1, the viability of *S. thermophilus* in yogurt formulations containing 0%, 1% and 2.5% chickpea flour was relatively stable across the five-weeks storage period. The viable count for yogurt with 5% chickpea flour remained lowest during storage. Although the viability of *L. delbrueckii* subsp. *bulgaricus* remained stable over the first two weeks, a significant decline in the viable count was observed in all yogurt formulations at week 3 (Figure 1). The largest viability drop was found in yogurt with 5% chickpea flour, followed by 2.5%. The viable count of *L. delbrueckii* subsp. *bulgaricus* decreased to approximately 7 log CFU/g in all formulations by the end of storage. Several studies reported similar findings, with higher viability of *S. thermophilus* compared to that of *L. delbrueckii* subsp. *bulgaricus* during yogurt storage [34,35]. The use of plastic containers for yogurt storage in this study, which may facilitate oxygen penetration into the food matrix [24], may be causally implicated in these results. Although both starter cultures are facultative anaerobes, *S. thermophilus* is also regarded as microaerophilic, tolerating minute amounts of oxygen. Therefore, the increase in oxygen penetration caused by the plastic packaging material has no significant impact on the viable count of *S. thermophilus.* In contrast, the oxygen permeability of the packaging may negatively affect the growth of *L. delbrueckii* subsp. *bulgaricus*. Additionally, bacteriocins produced by *L. acidophilus* LA5 in the presence of sugars are reported to be detrimental to *L. delbrueckii* subsp. *bulgaricus* [36]. Hence, the inclusion of *L. acidophilus* LA5 in this study may have caused a greater detrimental effect on *L. delbrueckii subsp. bulgaricus* than *S. thermophilus*.

The data in Figure 1 show that the lowest viability of both starter cultures was observed in yogurt fortified with 5% chickpea flour. This finding could be mainly attributed to the high acid stress in yogurts, which inhibits the growth of bacteria by acidifying the cytoplasm and inhibiting enzymatic reactions [36]. Concordant results were reported by Conway et al. [37], wherein *S. thermophilus* and *L. delbrueckii* subsp. *bulgaricus* were less tolerant of acids. In the present study, yogurt with 5% chickpea flour had a significantly higher acidity among all formulations. This may have resulted in a negative impact on the viability of starter cultures.

Although the viability of both probiotic bacteria in the yogurt decreased significantly (*p* < 0.05) during storage, the ultimate probiotic count in all yogurt formulations exceeded the minimum therapeutic value of 6 log CFU/g. As shown in Figure 1, the trend toward decreased viability of *L. acidophilus* LA5 across all formulations was very similar. However, yogurts with chickpea flour were found to have a clearly lower probiotic viability than plain yogurt from week 2, despite a temporary increase in the 2.5% chickpea flour formulation during week 4. However, *L. acidophilus* LA5 in all yogurt formulations had similar viability of around 7.40 log CFU/g at the end of storage.

Previous studies have implicated several factors in the viability of *L. acidophilus* LA5, including the type of milk and starter culture used in yogurt manufacture [38,39]. For example, Drakoularakou et al. [38] observed better growth of *L. acidophilus* LA5 in ovine milk as compared to bovine and caprine milk when incubated for 12 h at 37 °C. Moreover, the use of *L. delbrueckii* subsp. *bulgaricus* as a starter culture has detrimental effects on *L. acidophilus* LA5 because it can produce hydrogen peroxide, which is injurious to probiotic species [39]. Therefore, the decline in the viability of *L. acidophilus* LA5 observed in the current study was not surprising. Nevertheless, previous studies indicated that *L. acidophilus* can survive better in acidic conditions [40]. This is because *L. acidophilus* has the ability to resist changes in cytoplasmic pH due to its high cytoplasmic buffering capacity and membrane H^+^ conductance [41]. In this study, despite increased acidity associated with enrichment by chickpea flour, the pH of all yogurt formulations remained between 4 and 5, the optimal condition for *L. acidophilus* survival. For this reason, *L. acidophilus* LA5 in all yogurt formulations maintained satisfactory viability (>7 log CFU/g) to the end of storage.

Regarding *Bifidobacterium* BB12, a slight decrease within the first four weeks was observed in plain yogurt, while in the last week a significant reduction to 7.70 log CFU/g was recorded. However, the viable count of *Bifidobacterium* BB12 in all yogurts containing chickpea flour decreased by more than 1 log CFU/g during the first week. The probiotic viability in yogurts with 1% and 5% chickpea flour then remained constant during the later stages of storage. The 2.5% chickpea flour formulation showed a slight increase at week 2 and then remained stable. Although the viability of *Bifidobacterium* BB12 maintained a high level (>7 log CFU/g) in all yogurt samples to the end of storage, the viable count in yogurt with 5% chickpea flour was significantly (*p* < 0.05) lower than in the other formulations.

Higher acidity is detrimental to the growth of *Bifidobacterium* [24]. It has been reported that the optimum pH for the growth of *Bifidobacterium* ranges from 6 to 7, which is higher than that of the yogurt in the current study [42]. As a result, a large number of free hydrogen ions damaged the probiotic cells by disrupting the mass transfer through the cell membrane, leading to a decrease in probiotic viability. In addition, the increased concentration of undissociated organic acids, due to the addition of chickpea flour, aggravated the bactericidal effect, which provides an explanation for the larger reduction in bifidobacteria in yogurts containing chickpea flour, compared to that of plain yogurt [43]. Further, the changes in the microstructure of yogurt, due to the differing fat contents, may provide an unsuitable environment for probiotic growth [23]. For example, Vinderola et al. [44] indicated that full-fat yogurt had inhibitory effects on the growth of probiotics, compared to reduced-fat yogurt. Due to the use of various concentrations of chickpea flour in the present study, changes in microstructure and the composition of yogurt are possible. Such changes may have resulted from unfavorable conditions for bifidobacteria and have also contributed to the reduced viable count of bifidobacteria in yogurt fortified with chickpea flour compared to the plain yogurt. Nevertheless, the presence of yogurt starters is beneficial to the survival of *Bifidobacterium.* For instance, the proteolytic properties of *L. delbrueckii* subsp. *bulgaricus* provide amino acids essential to the growth of *Bifidobacterium* [45]. Moreover, *S. thermophilus* can act as an oxygen scavenger, which further improves the survival of strictly anaerobic *Bifidobacterium* BB12 [40]. Thus, the viability of bifidobacteria (>7 log CFU/g) was maintained at a satisfactory level in all formulations in the current study.

Accordingly, in terms of the viability maintenance of the two probiotic bacteria *L. acidophilus* LA5 and *Bifidobacterium* BB12, the addition of chickpea flour has no significant (*p* > 0.05) positive impact in yogurt formulations.

### 3.3. Probiotic Viability during Simulated Gastric and Intestinal Digestion

As yogurt starter cultures do not possess biological mechanisms to permit satisfactory gastrointestinal survival, the viable number during simulated gastrointestinal transit was assessed only for probiotic *Bifidobacterium* BB12 and *L. acidophilus* LA5 in the present study. The viability of both *L. acidophilus* LA5 and *Bifidobacterium* BB12 in all yogurt samples during simulated gastric digestion showed a significant decline (*p* < 0.05), though to different degrees (Table 3). The viability of *Bifidobacterium* BB12 in plain yogurt was less than 1 log CFU/g at the end of 180 min of digestion. Higher viable numbers were still noted at 180 min in yogurt formulations containing 1% and 2.5% chickpea flour: viable numbers were 4.60 and 4.30 log CFU/g, respectively. In yogurt samples containing 5% chickpea flour, relatively satisfactory viability was maintained only up to 60 min of exposure. Then, there was a dramatic decrease from 4.09 to less than 1 log CFU/g at the end of 180 min of exposure. *L. acidophilus* LA5 demonstrated a significantly higher gastric tolerance than *Bifidobacterium* BB12 in all yogurt samples (*p* < 0.05). The largest decrease in the viability of *L. acidophilus* LA5 was noted in yogurt containing 2.5% chickpea flour, which fell from 8.30 to 4.95 log CFU/g at the end of 180 min of digestion. All other samples maintained significantly higher viability at the end of gastric digestion (*p* < 0.05). Interestingly, the plain yogurt with no added chickpea flour demonstrated satisfactory protection of probiotic species during simulated gastric digestion.

As shown in Table 4, when exposed to simulated intestinal juices without bile salt, the viability of *Bifidobacterium* BB12 decreased significantly within an hour (*p* < 0.05). The viable counts in all formulations decreased to less than 1 log CFU/g at the end of 240 min. *L. acidophilus* LA5 demonstrated significantly higher viable numbers in all yogurts (>4.47 log CFU/g) at the end of digestion as compared to *Bifidobacterium* BB1 (*p* < 0.05). 

When yogurt samples were exposed to simulated intestinal juices with 0.3% bile salts, the two probiotics showed similar trends in the maintenance of viability. The viable counts of both probiotics in plain yogurts were reduced by more than 8.00 log CFU/g within 60 min. All yogurts containing chickpea flour maintained a relatively high value (>4.50 log CFU/g) until 60 min. At the end of 240 min of exposure, both probiotics in all formulations demonstrated poor viable numbers of less than 1 log CFU/g.

In order to achieve a therapeutic effect for consumers, probiotics must survive in the harsh conditions of the gastrointestinal tract. When probiotics pass through the stomach, the low pH and the antimicrobial property of pepsin are the main detrimental factors affecting the survival of these bacteria [46]. In the current study, the addition of 1% and 2.5% chickpea flour to yogurt demonstrated an enhanced protective impact on the viability of *Bifidobacterium* BB12 as compared to plain yogurt. The protective effect may be due to the relatively higher fat content of chickpea flour, which increased the buffering capacity of the yogurts. With increased fat content, the sensitivity of *Bifidobacterium* BB12 to the high acid environment was reduced significantly, resulting in higher viability after simulated gastric digestion. However, the formulation containing 5% chickpea flour only had a protective effect for the first 60 min. This could be attributed to the possible microstructural changes in the yogurt due to the addition of a higher concentration of chickpea flour, which may have exposed bifidobacteria to gastric juices directly during prolonged exposure to gastric juices. However, this phenomenon warrants further study. The addition of chickpea flour showed no significant protection of *L. acidophilus* LA5 in this study. Gastric juice tolerance of probiotics is strain-specific and, as mentioned above, *L. acidophilus* has a high tolerance to acidic environments, which may explain its high viability during simulated gastric digestion in all yogurt samples.

Generally, the intestinal environment is even more challenging to probiotic survival and colonization, as they are exposed not only to highly alkaline conditions (pH around 8.0), but also to bile [47]. In this study, bile salt demonstrated significantly negative impacts on the viability of probiotics, mainly due to the antimicrobial properties of bile salts. Accumulating in the cytoplasm, bile salts may cause protein aggregation, resulting in disruption of homeostasis and eventual cell death [48]. However, the addition of chickpea flour significantly increased bile tolerance in both probiotics. Similar protective effects have been reported for other legume and cereal products [49,50]. De Boever et al. [49] developed fermented milk with soy germ powder and observed improved viability of *L. reuteri*, with resistance to intestinal juices and bile salts. Additionally, Patel at al. [50] reported positive effects of malt, wheat and barley extracts on the bile tolerance of *Lactobacillus*. The protective effects of these ingredients on probiotics are mainly attributed to the physical protection they provide [51]. Moreover, some legume proteins, such as soy protein, may have the ability to bind bile salts, thereby reducing the damage caused to probiotics [52]. In the current study, the addition of chickpea flour provided a high concentration of additional protein, which may have sequestered some bile salts while also creating a physical barrier for probiotics against the intestinal environment. Therefore, in yogurts containing chickpea flour, both probiotics exhibited higher viability during 60 min of exposure to bile. However, there was no considerable difference in the viability of probiotics noted for differing levels of chickpea flour. Thus, future studies are needed to explore the mechanism underpinning this phenomenon.

In addition, further experiments are needed to evaluate the organoleptic acceptance of chickpea flour-enriched probiotic yogurt products.

## 4. Conclusions

In this study, the effect of chickpea flour enrichment on probiotic yogurt was evaluated in terms of the physicochemical properties of the product, microbial viability during five weeks of refrigerated storage and in vitro gastrointestinal transit tolerance of the probiotics. The formulation of yogurts incorporating chickpea flour to a concentration of 5% improved certain physicochemical properties, such as water holding capacity and susceptibility to syneresis. The viability of probiotic *Bifidobacterium* BB12 and *L. acidophilus* LA5 in all yogurt samples was satisfactory (>7 log cuf/g) during shelf-life. The addition of chickpea flour did not confer any additional advantage in maintaining the probiotic viability of yogurts during storage. The addition of 1% and 2.5% chickpea flour showed significant protective effects on *Bifidobacterium* BB12 during the simulated gastric digestion. Further, the addition of chickpea flour demonstrated a significant positive impact on probiotic survival when exposed to simulated intestinal juices with 0.3% bile salts. Therefore, chickpea flour may be used as a suitable ingredient to improve some physicochemical properties and the functional efficacy of certain probiotics during gastrointestinal transit.

## Figures and Tables

**Table 1:** Physiochemical properties of probiotic yogurt fortified with chickpea flour during first week of storage at 4 °C (*n* = 3, mean ± SD).

Formulation Chickpea Flour	pH	TA (%)	Total Solids (%)	Ash Content (%)	Water Holding Capacity (%)	Viscosity (cP)	Susceptibility to Syneresis (%)
0%	4.47 ± 0.12 ^a^	0.78 ± 0.02 ^a^	12.33 ± 0.35 ^a^	0.77 ± 0.02 ^a^	54.00 ± 3.46 ^a^	3333.33 ± 0.00 ^a^	49.00 ± 1.73 ^a^
1%	4.48 ± 0.10 ^a^	0.78 ± 0.02 ^a^	12.83 ± 0.01 ^a^	0.81 ± 0.02 ^b^	52.66 ± 1.99 ^a^	5333.33 ± 0.00 ^b^	45.50 ± 1.51 ^b^
2.5%	4.46 ± 0.07 ^a^	0.83 ± 0.03 ^b^	14.13 ± 0.17 ^b^	0.93 ± 0.02 ^c^	57.50 ± 4.99 ^b^	6499.95 ± 288.22 ^c^	44.50 ± 1.51 ^b^
5%	4.44 ± 0.09 ^a^	0.87 ± 0.03 ^c^	15.64 ± 0.17 ^c^	1.09 ± 0.17 ^d^	58.14 ± 3.08 ^b^	7499.95 ± 288.22 ^d^	39.00 ± 1.73 ^c^

^a, b, c, d^ Values in the same column having different superscripts differ significantly (*p* < 0.05).

**Table 2:** Color of probiotic yogurt fortified with chickpea flour during first week of storage at 4 °C (*n* = 3, mean ± SD).

Formulation Chickpea Flour	Color		
	L *	a *	b *
0%	61.35 ± 8.91 ^a^	−0.95 ± 0.19 ^a^	5.65 ± 4.23 ^a^
1%	62.60 ± 2.60 ^a^	−1.30 ± 0.56 ^b^	5.65 ± 4.23 ^ac^
2.5%	60.4 5± 4.24 ^a^	−1.15 ± 0.61 ^ab^	6.80 ± 0.69 ^ab^
5%	62.95 ± 0.09 ^a^	−1.00 ± 0.09 ^a^	6.45 ± 3.71^c^

^a, b, c, d^ Values in the same column having different superscripts differ significantly (*p* < 0.05).

**Table 3:** Effect of simulated gastric juices (pH 2.0) on the viability of probiotics in yogurt formulations containing chickpea flour during 180 min of exposure (viable counts shown as log CFU/g, *n* = 2).

Probiotics	Formulation Chickpea Flour	Time (min) during the in Vitro Gastric Digestion			
		0	1	60	180
*Bifidobacterium*	0%	8.33 ± 0.20 ^a^	8.72 ± 0.42 ^a^	<1	<1
	1%	8.31 ± 0.13 ^a^	8.54 ± 0.39 ^a^	4.70 ± 0.00 ^b^	4.60 ± 0.00 ^b^
	2.5%	7.54 ± 0.39 ^a^	8.27 ± 0.17 ^b^	4.60 ± 0.00 ^c^	4.30 ± 0.00 ^d^
	5%	8.20 ± 0.80 ^a^	8.13 ± 0.03 ^a^	4.09 ± 0.00 ^b^	<1
*L. acidophilus*	0%	8.30 ± 0.15 ^a^	8.12 ± 0.07 ^a^	5.86 ± 0.25 ^b^	5.69 ± 0.00 ^b^
	1%	7.85 ± 0.56 ^a^	8.00 ± 0.01 ^a^	6.05 ± 0.63 ^b^	5.62 ± 0.11 ^b^
	2.5%	8.30 ± 0.03 ^a^	8.07 ± 0.11 ^a^	5.86 ± 0.14 ^b^	4.95 ± 0.00 ^c^
	5%	8.06 ± 0.03 ^a^	7.94 ± 0.12 ^a^	6.02 ± 0.54 ^b^	5.69 ± 0.00 ^b^

^a, b, c, d^ Mean values within the same row followed by different superscript letters represent a significant difference to that at 0 min (*p* < 0.05).

**Table 4:** Effect of simulated intestinal juices (with or without 0.3% bile salt, pH 8.0) on the viability of probiotics in yogurt formulations containing chickpea flour during 240 min of exposure (viable counts shown as log CFU/g, *n* = 2).

Probiotics	Bile Salt	Formulation Chickpea Flour	Time (min) during the in Vitro Intestinal Digestion			
			0	1	60	240
*Bifidobacterium*	0%	0%	9.16 ± 0.25 ^a^	9.12 ± 0.22 ^a^	4.70 ± 0.00 ^b^	<1
		1%	8.90 ± 0.16 ^a^	8.74 ± 0.09 ^a^	3.00 ± 0.00 ^b^	<1
		2.5%	8.93 ± 0.29 ^a^	8.95 ± 0.31 ^a^	5.17 ± 0.00 ^b^	<1
		5%	8.95 ± 0.36 ^a^	8.84 ± 0.30 ^a^	4.84 ± 0.00 ^b^	<1
	0.3%	0%	9.06 ± 0.48 ^a^	8.90 ± 0.39 ^a^	<1	<1
		1%	8.84 ± 0.41 ^a^	8.92 ± 0.50 ^a^	5.20 ± 0.00 ^b^	<1
		2.5%	8.48 ± 0.46 ^a^	8.32 ± 0.26 ^a^	5.00 ± 0.00 ^b^	<1
		5%	8.74 ± 0.53 ^a^	8.71 ± 0.55 ^a^	4.80 ± 0.00 ^b^	<1
*L. acidophilus*	0%	0%	8.78 ± 0.26 ^a^	8.36 ± 0.16 ^b^	5.54 ± 0.55 ^c^	4.77 ± 0.00 ^d^
		1%	8.69 ± 0.29 ^a^	8.38 ± 0.03 ^b^	6.14 ± 0.79 ^c^	4.47 ± 0.00 ^d^
		2.5%	8.69 ± 0.25 ^a^	8.70 ± 0.31 ^b^	5.62 ± 0.18 ^b^	4.95 ± 0.00 ^c^
		5%	8.52 ± 0.45 ^a^	8.07 ± 0.01 ^a^	5.43 ± 0.16 ^b^	4.60 ± 0.00 ^c^
	0.3%	0%	8.30 ± 0.15 ^a^	8.07 ± 0.17 ^a^	<1	<1
		1%	7.85 ± 0.56 ^a^	7.60 ± 0.54 ^a^	5.13 ± 0.16 ^b^	<1
		2.5%	8.30 ± 0.03 ^a^	7.94 ± 0.32 ^a^	5.25 ± 0.07 ^b^	<1
		5%	8.06 ± 0.03 ^a^	7.53 ± 0.30 ^a^	4.60 ± 0.00 ^b^	<1

^a, b, c, d^ Mean values within the same row followed by different superscript letters represent a significant difference to that at 0 min (*p* < 0.05).
